# Cytokine quantification and association with cervical length in a prospective cohort of pregnant women

**DOI:** 10.1186/s12884-023-05776-2

**Published:** 2023-06-20

**Authors:** Helmer Herren, Alessandra C. Marcolin, Marco A. Barbieri, Heloisa Bettiol, Viviane C. Cardoso, Silvana M. Quintana, Ricardo C. Cavalli

**Affiliations:** 1 Department of Gynecology and Obstetrics, Ribeirão Preto Medical School University of São Paulo, Av. Bandeirantes, 3900 - 8º Andar – HCRP Campus Universitário - Ribeirão Preto – SP CEP: 14049-900, São Paulo, Brazil; 2 Department of Puericulture and Pediatrics, Ribeirão Preto Medical School University of São Paulo, Ribeirao Preto, Brazil

**Keywords:** Preterm birth, Prematurity, Chemokines, Cervical length, Short cervix, Inflammatory markers, Biomarkers, A transvaginal ultrasound, Preterm inflammatory pathways, Preterm anti-inflammatory pathways

## Abstract

**Background:**

Preterm birth is a leading cause of infant morbidity and mortality; its multifactorial causes are an obstacle to understanding etiology and pathogenesis. The importance of cytokines and inflammation in its etiology and association with the short cervix is nowadays well-proven. To date, there are no reliable biological or biochemical markers to predict preterm birth; even though the length of the cervix has high specificity, its sensitivity with the cervix below 2.5 cm is low.

**Objective:**

We study the association of plasma cytokine levels and cervical length in search of predictors of preterm birth.

**Study design:**

We evaluated a total of 1400 pregnant women carrying a single fetus between 20 and 25 weeks of gestation, and 1370 of them after childbirth in a nested case–control study of a prenatal cohort. Eligible pregnant women were interviewed and submitted to obstetric morphological and transvaginal ultrasound with cervical length measurement, gynecological examination, and blood collection. Preterm birth occurred in 133 women, 129 included in the study, and a control group randomly selected at a 2:1 ratio. A total of 41 cytokines with a higher probability of being associated with preterm birth or being of significance during labor were determined.

**Results:**

Cytokine and cervical length analysis by multivariate analysis of the conditional interference tree revealed that growth-related oncogene values of less than 2293 pg/mL were significantly associated with a cervical length of less than 2.5 cm.

**Conclusions:**

As well as a cervical length shorter than 2.5 cm, growth-related oncogene levels of less than 2293 pg/ml may be associated with an increased risk of PB. Analysis based on the association of biomarkers and of the interaction between cytokines is a promising pathway in search of a predictor of preterm birth.

## Condensation

A biochemical marker associated with a short cervix that allows earlier and more specific selection of patients at risk of preterm birth is relevant.

## Introduction

Preterm birth (PB) is a challenge in obstetrical practice. An estimated 15 million babies worldwide are born too early every year [1], and approximately 1 million children die each year due to complications of preterm birth [2]. In the United States, in 2018, the prevalence of PB reached 10.02% of all liveborn [3]. In Brazil, the incidence of PB reached a rate of 14.7% in 2019—SINASC, 2019 [4].

The current consensus in the literature is that the mechanisms involved in PB are multifactorial. The change from quiescent myometrium to a contractile one involves a change in signaling between the anti-inflammatory and proinflammatory pathways, with the activation of chemokines, cytokines, and metalloproteinases. A single inflammatory pathway is probably activated both in term and preterm births via an innate immune system. During the acute phase, this process is due to the activation of receptors that recognize pathogen-associated molecular patterns (PAMPs). During the chronic phase, it based on the activation of the receptor for advanced glycation end products (RAGE) [5] recognize damage-associated molecular patterns (DAMPs) [6, 7]. The secreted cytokines, in turn, stimulate the synthesis of metalloproteinases and prostaglandins, thus inducing uterine contractions, cervical maturation and rupture of the membranes.

Several studies have demonstrated a strong association between a high concentration of inflammation biomarkers and a short cervix [8] and may be caused by increased cytokine levels that initiate the break of connective tissue in the cervix [9]. Alternately, a short cervix may facilitate ascending uterine infections that result in increased cytokine levels [10]. Another pathway could be a common cytokine-mediated factor that would trigger cervix shortening and PB [8].

Evaluation of the cervix by transvaginal ultrasound is today considered with predictive value for the detection of pregnant women at high risk of PB [11]. The cervical length is related to exposure and outcome, but mainly by being part of the causal chain that links exposure to the outcome, it can be considered a variable that mediates the effects of cytokines on PB. The cervical fading causing cervical shortening precedes by five to six weeks the onset of both term labor and PB [12].

Rozenberg (2017) pointed out that the predictive value of a short cervix is 17.8% when considering a PB prevalence of 4.3%, which means that if this cut-off point is applied, most women with a short cervix and no history of PB will give birth at term. Thus, it is justified to look for a biomarker that will improve the predictive value of a short cervix for PB [13].

On this basis, the objective of the present study was to look for cytokines and chemokines’ associations with a short cervix that could be candidates for biomarkers and improve the predictive value of the measurement of cervix length alone.

## Material and methods

The present study was a case–control nested in a convenience cohort approved by the Research Ethics Committee of The University Hospital, Faculty of Medicine of Riberão Preto, University of São Paulo (HCFMRP-USP; protocol number: 4116/2008). The pregnant women recruited from two Brazilian cities, São Luís, state of Maranhão, and Ribeirão Preto, state of São Paulo, between February 2010 and February 2011. The present analysis conducted on the Ribeirão Preto cohort and recruitment performed in hospitals and health units of the municipal health network. A total of 1400 pregnant women evaluated during the second trimester of gestation, and 1370 evaluated postpartum in the maternities of Ribeirão Preto at the time of childbirth.

Inclusion criteria were healthy pregnant women with a single fetus, between 20 to 25 weeks of gestation. The last menstrual period (LMP) established the gestational age when accordant with the ultrasound done before 20 weeks. If discordant we used ultrasound gestational age. Exclusion criteria were fetuses with major congenital malformations or chromosome syndromes diagnosed before 20 weeks of gestation. All patients signed written informed consent to participate in the study.

**Fig. 1:**
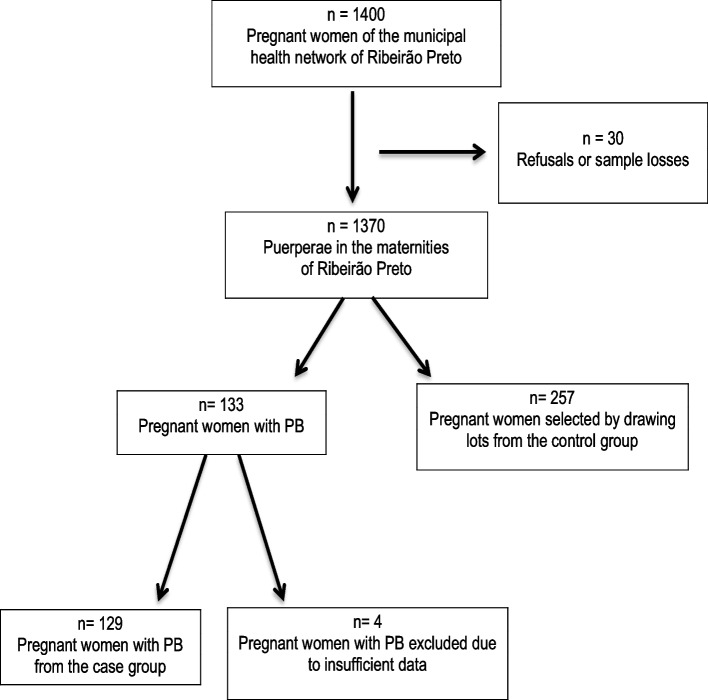
Flow diagram of the pregnant women participating in the cohort

The women selected were interviewed and submitted to exams, including obstetric ultrasound, gynecological examination, and blood collection between 20 and 25 weeks of gestation. The patient’s selection is summarized in a flowchart (Fig. 1).

We calculated the sample size and the power of the study based on the reported prevalence of explanatory variables. Due to the fact that this was a case–control study nested in a broader cohort with a convenience sample, the sample size was calculated in the base project [14].

The ultrasound exams were carried out by two trained observers advised about the methods always using the same equipment (Model HDI 11, GE Voluson 730), with a 5–9 MHz transvaginal transducer. The cervix length and its biophysical aspects were assessed according to the criteria described by TO et al. in 2001 [15]. The cervix cut-off for the assessment of the association of plasma cytokines with PB was ≤ 2.5 cm [11].

The gynecological examination made for the detection of bacterial vaginosis used the NUGENT > 7 criterion or presence of clue cells for the diagnosis.

Venous blood collected for the determination of plasma cytokines and chemokines used a high sensitivity kit (Milliplex Map Human Cytokine/Chemokine. Cat HCYTOMAG-60 K-PX41 Millipore Corporation, Billerica, MA, USA). Plasma centrifuged at 10,000 rpm for 10 min, and the technique was carried out according to manufacturer recommendations, with analyte quantitation using a Luminex 200 instrument (Millipore Corporation, Billerica, MA, USA). We selected 41 cytokines whose presence in maternal blood was frequently associated with PB or to be important for labor in previous studies [16, 17]. The cut-off value above the 95th percentile was considered altered in the sample analyzed for association with PB.

Pregnant women who gave birth before 37 weeks of gestational age were considered cases. We monitored confounding variables that might influence the number of PB and maternal inflammation in the cohort. The following data were obtained at childbirth: hospital and date of birth, gestational age at birth in days, birth weight, and time of maternal amenorrhea.

The continuous quantitative variables, such as maternal age and parity, were divided into ranges. Maternal age divided into three groups: < 19, 19–35 and > 35 years, a division justified by the fact that adolescent pregnant women and older pregnant women face specific risks and peculiarities. The parity was also divided into three groups: primiparae, 2 to 3 childbirths, and multiparae. Again, the specific risks and peculiarities of each group justify the stratification.

We defined discrete qualitative variables, such as smoking habits and the history of PB, as that associated with any quantity or frequency of smoking during the various gestational periods, any type of smoking cigarette. The occurrence of previous PB before 37 weeks of gestation was not stratified and established as a PB.

We performed exploratory data analysis by measurements of central position and dispersal, and the qualitative variables described in terms of absolute and relative frequencies. Univariate analysis was carried out to determine the factors associated with the outcome (PB), and the results analyzed by the nonparametric Mann–Whitney test (or Wilcoxon test for independent samples) and the chi-square test.

We used the conditional inference tree to determine the factors associated with PB. The analysis used the R software version 3.3.3. The univariate relationship between cytokines and outcome was first determined, followed by the construction of two additional models, one of them considering as covariables the cytokines that were relevant in univariate analysis and cervical length, and the other involving all cytokines plus the cervical length variable.

## Results

PB occurred in 133 of the 1370 pregnant women included in the study. Due to the loss of 4 women to follow-up, the case group consisted of 129 women, with a 9.4% prevalence of PB. The case and control groups did not differ significantly in parity, maternal age, or mean BMI, as shown in Table 1.

**Table 1:** Age, parity, and body mass index of the case and control groups

Variable	Cases (*n* = 129)	Controls (*n* = 257)	*P*
Maternal age (N. %)			0.5766
< 19	13 (10.1)	20 (7.8)	
19—35	104 (80.6)	218 (84.8)	
> 35	12 (9.3)	19 (7.4)	
Parity (N. %)			0.1966
1	48 (37.2)	110 (42.8)	
2—3	57 (44.2)	116 (45.1)	
≥ 4	24 (18.6)	31 (12.15)	
Mean BMI (SD)	26.1 (5.7)	26.8 (4.7)	0.1500

*BMI* Body mass index, *P* P valor, *SD* Standard deviation

There was an association between PB and maternal smoking history during the present gestation, with a more significant proportion of PB, as expected. However, the occurrence of genitourinary infection (bacterial vaginosis and urinary infection) during pregnancy was not associated with the outcome of our study (Table 2).

**Table 2:** Association of previous preterm birth (PB), maternal smoking during pregnancy, and genitourinary infection with preterm birth

Variable	Cases (*n* = 129)	Controls ^a^ (*N* = 257)	*P*
Previous PB (N, %)			< 0.0001
Yes	87 (67.4)	18 (6.9)	
No	42 (32.6)	237 (92.9)	
Maternal smoking during pregnancy (N, %)			0.0014
Yes	46 (35.6)	53 (20.6)	
No	83 (64.4)	202 (79.3)	
Genitourinary infection (N, %)			0.5772
Yes	35 (27.1)	63 (24.5)	
No	94 (72.9)	194 (75.5)	

*P* P valor

^a^ The variation in number and percentages in some categories was due to exclusion of ignored results

When the dependent variables were related to the PB outcome, the univariate logistic regression model’s application revealed that 14 cytokines, cervical length, and newborn age differed significantly between the PB and the control groups. Five of these 14 cytokines classified as inflammatory (IFN-A2, GRO, IL1alpha, MIP-1ª, TNFB), five as an anti-inflammatory (IL10, SCD40L, IL-1RA, IL1B, IL4), and four as having both functions (Flt-3 l, PDGF-AA, PDGF-BB, IL15). The mean values detected were surprising lower in the case group than in the control group (Table 3).

**Table 3:** Dependent variables that showed a significant difference between the PB group and control. The cytokines in italics are proinflammatory, the underlined ones are anti-inflammatory, and those presented in normal writing may have the two functions

Variable	Group	N	Mean	Sandard Deviation	Median	Minimum	Maximum	*P*-value
**GA at delivery (days)**	**Case (PB)**	**129**	**241.59**	**20.56**	**247**	**166**	**282**	** < 0.001**
	**Control (FT)**	**257**	**276.65**	**8.45**	**276**	**259**	**300**	
**Cervical length**	**Case**	**128**	**3.36**	**0.74**	**3.36**	**0.77**	**5.2**	**0.00214**
	**Control**	**257**	**3.62**	**0.7**	**3.6**	**1.8**	**6.3**	
**Flt-3L**	**Case**	**129**	**65.65**	**66.57**	**48.38**	**0**	**540.9**	**0.01227**
	**Control**	**257**	**92.16**	**124.68**	**62.9**	**0**	**1334**	
***IFN-A2***	**Case**	**129**	**78.27**	**57.28**	**69.35**	**0**	**217.52**	**0.0442**
	**Control**	**257**	**94.64**	**68.55**	**71.64**	**0.06**	**367.77**	
***GRO***	**Case**	**129**	**1599.91**	**808.87**	**1442**	**495.33**	**5528**	** < 0.001**
	**Control**	**257**	**2090.28**	**1331.3**	**1730**	**281.29**	**10,262**	
**IL-10**	**Case**	**129**	**12.6**	**13.45**	**8.9**	**2.44**	**115.37**	**0.048**
	**Control**	**257**	**20.2**	**65**	**10.55**	**2.3**	**846.41**	
**PDGF-AA**	**Case**	**129**	**8134.91**	**6529.58**	**6288**	**1150**	**31,699**	**0.01677**
	**Control**	**257**	**10,154.54**	**8118.97**	**7489**	**788.75**	**55,580**	
**PDFG-BB**	**Case**	**129**	**26,199.68**	**11,017.64**	**24,047**	**8252**	**74,017**	**0.01059**
	**Control**	**257**	**28,832.49**	**11,748.34**	**27,425**	**1555**	**59,251**	
**SCD40L**	**Case**	**129**	**314,901.64**	**268,159.23**	**209,980**	**21,889**	**1,303,900**	**0.009587**
	**Control**	**257**	**375,637.95**	**316,068.24**	**268,806**	**1928**	**1,712,141**	
**IL-1RA**	**Case**	**129**	**110.68**	**108.62**	**76.02**	**6.25**	**743.25**	**0.001443**
	**Control**	**257**	**149.72**	**296.33**	**89.59**	**6.25**	**4469**	
***IL-1alpha***	**Case**	**129**	**51.37**	**60.07**	**25.43**	**1.25**	**352.74**	**0.005089**
	**Control**	**257**	**72.79**	**101.15**	**41.08**	**1.25**	**883.88**	
**IL-1B**	**Case**	**129**	**6.28**	**5.04**	**4.27**	**0.87**	**30.26**	**0.01286**
	**Control**	**257**	**8.24**	**14.52**	**5.28**	**1.3**	**191.95**	
**IL-4**	**Case**	**129**	**56.8**	**98.36**	**12.94**	**0.11**	**319.34**	**0.03508**
	**Control**	**257**	**67.87**	**107.09**	**17.25**	**0.41**	**711.74**	
***MIP-1A***	**Case**	**129**	**18.35**	**25.43**	**13.12**	**1.91**	**262.78**	**0.03645**
	**Control**	**257**	**37.59**	**126.5**	**14.43**	**1.54**	**1363**	
***TNF-B***	**Case**	**129**	**29.72**	**40.23**	**15.8**	**1.23**	**200.45**	**0.02803**
	**Control**	**257**	**41.73**	**85.22**	**20.57**	**1.72**	**1040**	
**IL-15**	**Case**	**129**	**19.64**	**50.74**	**6.12**	**2.17**	**272.93**	**0.001117**
	**Control**	**257**	**20.03**	**48.25**	**9.05**	**1.86**	**350.14**	

*Abbreviations: GA* Gestational age, *PB* Preterm birth, *FT* Full term birth, *Flt-3L* FMS-like tyrosine kinase 3 ligand, *IFN-A2* Interferon alpha-2, *GRO* Growth-related oncogene, *IL-10* Interleukin-10, *PDGF-AA* Platelet Derived Growth Factor-AA, *PDFG-BB* Platelet Derived Growth Factor-BB, *SCD40L* Soluble CD40 ligand, *IL-1RA* Interleukin-1 receptor antagonist, *IL-1alpha* Interleukin 1-alfa, *IL-1B* Interleukin 1-beta, *IL-4* Interleukin-4, *MIP-1A* Macrophage Inflammatory Protein-1alpha, *TNF-B* Tumor necrosis factor-beta, *IL-15* Interleukin-5

Analysis of the association between cytokines and cervical length using the conditional inference tree revealed six cytokines (FlT3l, IFN.A2, PDGF.AA, PDFG.BB, IL.1alpha, and GRO), and cervical length showed a relevant difference between the case group and control. Three of these six cytokines are inflammatory, and three have both pro and anti-inflammatory functions.

**Fig. 2:**
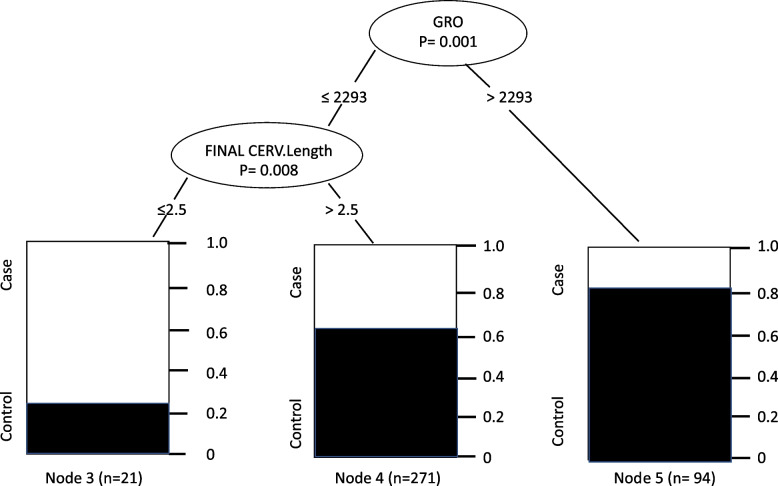
Results of multivariate analysis of the conditional inference tree considering as covariables the cytokines IFN.A2, PDGF.AA, PDFG.BB, IL.1alpha, GRO, and cervical length. Node 1 – Pregnant women classified according to a GRO value ≤ or > 2293 pg/ml, Node 2—Pregnant women with GRO ≤ 2293 pg/ml classified as having a cervical length ≤ or > 2.5 cm, Node 3 – Proportion of case and control pregnant women with GRO ≤ 2293 pg/ml and cervical length ≤ 2.5 cm, Node 4 – Proportion of case and control pregnant women with GRO ≤ 2293 pg/ml and cervical length > 2.5 cm, Node 5—Proportion of case and control pregnant women with GRO > 2293 pg/ml

However, multivariate analysis of the conditional inference tree considering these six cytokines as covariables and cervical length revealed that only GRO was significantly associated with a cervical length of less than 2.5 cm and PB when its value was below 2293 pg/ml (Fig. 2).

**Fig. 3:**
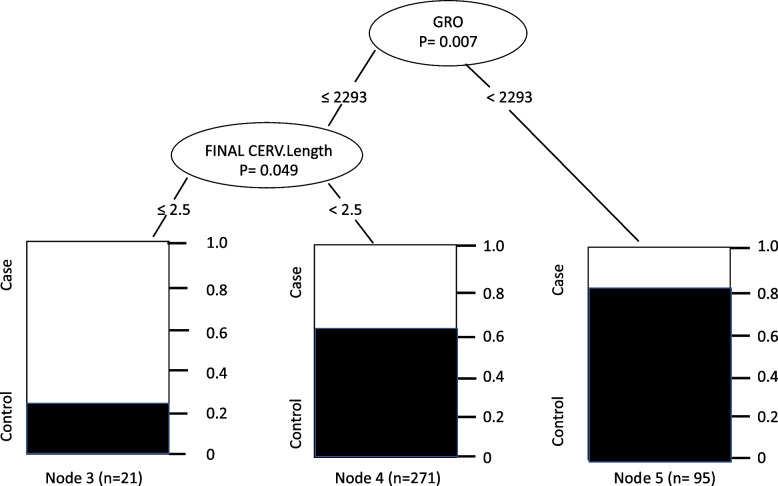
Results of multivariate analysis of the conditional inference tree considering as covariables all the cytokines understudy plus cervical length. Node 1 – Pregnant women classified according to a GRO value ≤ or > 2293 pg/ml, Node 2—Pregnant women with GRO ≤ 2293 pg/ml with a cervical length ≤ or > 2.5 cm, Node 3 – Proportion of case and control pregnant women with GRO ≤ 2293 pg/ml and cervical length ≤ 2.5 cm, Node 4 – Proportion of case and control pregnant women with GRO ≤ 2293 pg/ml and cervical length > 2.5 cm, Node 5—Proportion of case and control pregnant women with GRO > 2293 pg/ml

We had similar results when considering all the cytokines understudy as covariables plus cervical length. Again, a GRO value below 2293 pg/ml is associated with a cervical length of less than 2.5 and PB (Fig. 3).

## Structured discussion

### Main study findings

The proinflammatory cytokines classically associated with PB in the literature, such as IL-6 and IL-8, did not associate with PB or with short cervical length in this study. This finding in serum cytokines may have biological plausibility. Simhan et al., 2003 studying cervical proinflammatory cytokines and the risk of clinical chorioamnionitis in pregnant women, finding lower cervical levels of IL-1b, IL-6, and IL-8 associated with clinical chorioamnionitis knowingly related with PB [18]. Mean cytokine levels were higher in the control group than in the PB group. A significant difference in IL-10 was also observed by univariate analysis between the case and control groups, with significantly higher levels in the control group, confirming literature data that show a regulatory action for IL-10.

The present data showed that total GRO serous levels lower than 2293 pg/ml was significantly associated with a cervical length of ≤ 2.5 cm and PB. Six cytokines showed association with short cervix and PB in the univariate analysis of the conditional inference tree. Of these, five showed association with PB in the literature. One, IFN.A2, although involved in the inflammatory response, has not been reported to be related to PB. Out of these five cytokines, only GRO showed association with PB in multivariate analysis using the conditional inference tree. This fact deserves a more in-depth analysis, along with literature data, regarding this association with PB.

### The context of cytokines and chemokines mainly CXC family in PB

The literature has shown an association between increased serum proinflammatory cytokines and PB [17]. In studies on humans, the cytokines that showed a higher association with PB were IL-1, TNF, and IL-6. IL-6 seems to be an important marker of inflammation and predictor of PB [19]. Cytokines with an anti-inflammatory action play a regulatory role in the inflammatory response, and on the control of tissue injury due to inflammation. Within this context, IL-10, abundantly present in the placenta and gravid uterus, showed association with the regulation of inflammation in pregnancy, and some studies, specifically regarding PB, have reported a reduction of IL-10 [20].

Studies on animal models of IL-6 deficiency have shown an average 24 h delay in the triggering of parturition in deficient mice compared to mice without IL-6 deficiency and an increased resistance to PB induced by lipopolysaccharides [21]. Studies on IL-10-deficient mice have shown that these animals were more susceptible to PB induced by lipopolysaccharides [22]. There are evidences that IL-10-dependent regulation of TNF, IL-6, and IL-1 reduces the inflammatory response, also reducing the probability of PB induced by infection and inflammation [23]. These findings indicate that the interaction between pro and anti-inflammatory cytokines play an important role in childbirth and PB.

The CXC family is one of the two major subfamilies of chemokines and initially described as an endogenous growth factor of tumor cells in melanomas. It is produced by different cell types, such as synovial cells, monocytes, fibroblasts, and endothelial cells, and its production is directly influenced by IL-8, which is responsible for activating neutrophils and chemotaxis of inflammatory response cells [24].

GRO is a chemokine which consists of three subunits: GROα/CXCL1, GROβ/ CXCL2, and GROγ/CXCL3 produced by different cell types such as synovial cells, monocytes, fibroblasts, and endothelial cells its production are directly influenced by IL-8, which is responsible for neutrophil activation and the chemotaxis of cells of the inflammatory response [25]. Some studies showed that an increase in chemokines of the CXC family in maternal serum [26] and amniotic fluid of patients with chorioamnionitis [27] was associated with PB.

Chemokines appear to act in a network of interaction, guaranteeing a robust defense system whose protection continues functioning even when one of the links of the network is compromised [28]. Chemokines are known to be natural protease substrates. Regarding GRO, clues show that it is degraded and inactivated by the protease dipeptidyl peptidase-IV (CD26/DPP IV) through the CXCR2 receptor, with the activation of neutrophils of hematopoietic stem cells. In the case of GROγ, studies have shown activation by proteases but have not determined whether the effect observed was increased neutrophil activation and migration. King et al. (2000, 2001) reported that the GROβ fragments truncated by the proteinases have more affinity for, and selectively bind to CXCR2 receptors and are ten times more potent in neutrophil activation than intact GROβ [29, 30].

### The CXC family chemokines lower serum concentration and PB

Most of the studies on cytokines and chemokines analyzed on amniotic fluid, and few assessed the association of chemokines of the CXC family, mainly CXCL1/CXCL2/CXCL3, in maternal serum with PB. Aminzadeh et al. determined CXCL10 and CXL12 in the cord blood of newborns and maternal serum, and detected a significantly increased CXCL10 concentration in both the mothers and newborns of the PB group, while the concentration of CXL12 was significantly increased only in the cord blood of preterm newborns [26]. Laudanski et al. (2014) observed significantly lower total serum GRO concentrations in pregnant women with PB and with no signs of infection compared to women with term delivery. However, it was not possible to establish a causal relationship between the serum concentration of this cytokine and premature birth [31].

The serum value of total GRO detected in the present study was less than 2293 pg/ml in association with a short cervix and with PB. There is no obvious explanation in the literature about the reason for the reduction of some chemokine levels in pregnant women who progress to PB. The lower chemokine concentrations may predispose to chorioamnionitis, which is a possible cause of PB [18].

### Strengths and limitations

It is positive that the present study is nested in a prospective cohort, confounding variables evaluated in terms of the outcome with the reduction of information bias. The excluded pregnant women carrying twin fetuses permitted greater control of the confounding variables. Data collection between 20 and 25 weeks of gestation it is the ideal time for the detection of biomarkers of PB and measurement of cervical length [15].

The single determination of cytokines between the 20th and 25th week of gestation limited the conclusions; a longitudinal assessment of the interaction of biomarkers might have provided more relevant data. The late release of cytokines in the inflammatory pathway of labor and their association mainly with acute inflammation may also be a limitation for their use as a PB predictor.

Inclusion of all preterm deliveries, spontaneous or provider-initiated preterm deliveries, has a possible association with the occurrence of an error type II that can justify not finding a significant variation of cytokines between the control and case groups.

Several studies have shown that multiple factors influence the inflammatory load that, based on multiple and redundant biological clocks, will influence the triggering of labor and PB. The understanding of their interactions and associations in the search for biomarkers has already led to some progress in the prediction of PB. However, their detection is still late and does not permit the establishment of appropriate therapies for prematurity prevention.

## Conclusion

The present study shows a GRO value below 2293 pg/ml associated with a cervical length of less than 2.5 and PB. Our data suggest that the evaluation of GRO during the second trimester with cervical length represents a promising pathway in search of a predictor of PB.

## Data Availability

The datasets generated and/or analysed during the current study are not publicly available due to privacy and ethical restrictions but are available from the corresponding author on reasonable request.
